# Correlation between CCL2, CALCA, and CX3CL1 gene polymorphisms and chronic pain after cesarean section in Chinese Han women: A case control study

**DOI:** 10.1097/MD.0000000000016706

**Published:** 2019-08-23

**Authors:** Guoping Ma, Jingli Yang, Bange Zhao, Chengquan Huang, Rui Wang

**Affiliations:** a Department of Anesthesiology, Shanghai University of Medicine & Health Sciences Affiliated Zhoupu Hospital, Shanghai; b Department of Anesthesiology, The Affiliated Shanghai Pudong Hospital of Fudan University Shanghai; c Department of Anesthesiology, HanDan Central Hospital, Hebei, China.

**Keywords:** CALCA, CCL2, CX3CL1, risk factor, single nucleotide polymorphism

## Abstract

**Objective:**

Postoperative chronic pain is characterized by high incidence, long duration, and complex pathogenesis. The purpose of this study was to investigate the correlation between the single nucleotide polymorphisms of the *CCL2* gene rs4586 (g.5974T>C), *CALCA* rs3781719 (−692T>C), *CX3CL1* rs614230 (2342C>T), and the risk of postoperative chronic pain in Chinese Han women.

**Methods:**

We analyzed the *CCL2* gene rs4586, *CALCA* rs3781719, *CX3CL1* rs614230 single nucleotide polymorphism (SNPs) of 350 Chinese Han women with chronic postsurgical pain (CPSP) 6 months after cesarean section and 350 healthy women without chronic pain (HC). The levels of CCL2, CALCA, and CX3CL1 in serum were detected by enzyme-linked immunosorbent assay (ELISA).

**Results:**

The *CCL2* rs4586 T allele and the *CX3CL1* gene rs614230C allele were protective factors for CPSP risk (adjusted OR = 0.766, 95% CI: 0.675–0.865 and OR = 0.336, 95% CI: 0.644–0.835). The *CALCA* gene rs3781719C allele was a risk factor for CPSP (adjusted OR = 1.273, 95% CI: 1.125–1.424). *CCL2* rs4586, *CX3CL1* gene rs614230, and *CALCA* gene rs3781719 locus gene polymorphisms were associated with serum CCL2, CX3CL1, and CALCA protein levels.

**Conclusion:**

Our results support that *CCL2* gene rs4586, *CALCA* rs3781719, *CX3CL1* rs614230 gene polymorphism are associated with the occurrence of chronic pain after cesarean section in Chinese Han women.

## 1 Introduction

Chronic postsurgical pain (CPSP) refers to the existence of pain associated with surgical-related pain for at least 2 months, except for other causes (such as chronic infection, recurrence of malignant tumor, etc).^[1]^ Open chest surgery, breast cancer (modified) radical operation, cesarean section, amputation, and so on are more likely to lead to CPSP, especially thoracotomy and amputation, the incidence of CPSP can be as high as 50% to 80%.^[2]^ Wang et al^[3]^ previous follow-up of 466 patients undergoing thoracotomy showed that the incidence of CPSP was as high as 64.5%, which seriously affected the quality of life of the patients after operation.

The mechanism of CPSP is complicated, the nerve injury caused by operation is the necessary premise of CPSP, and the excessive inflammatory counteraction of nervous system and abnormal immunomodulation play a key role in the transition from acute pain to CPSP after operation.^[4–6]^ Although the specific mechanism of central inflammatory response to CPSP induced by peripheral tissue and/or nerve injury is unclear, however, many studies have shown that central inflammatory response can enhance the nociceptive reflex arc of the spinal cord and induce central sensitization to nociceptive stimuli.^[7–10]^ When the peripheral nociceptors were sensitized by surgical injury, a large number of abnormal excitatory electrical activities were transmitted to the posterior horn of the spinal cord, which resulted in the abnormal opening of calcium channels in the presynaptic membrane and the enhancement of abnormal discharge.

The expression of CCL2 and its receptor CCR2 in the spinal cord and dorsal root ganglion (DRG) was increased by peripheral or central nerve injury. CCL2/CCR2 mediates glial cell-neuron interaction in central sensitization and chronic pain regulation during the neuroinflammatory response of peripheral nerves after surgical injury.^[11]^ Teler et al^[12]^ research shows that *CCL2* rs4586 gene polymorphism is associated with Gestational diabetes mellitus (GDM), the mechanism may be that the polymorphism of this locus affects the expression of CCL2 protein. Calcitonin-related polypeptide alpha (CALCA, α-CGRP) is a 37 amino acid neuropeptide with extensive biological effects and is one of the mediators of neurogenic inflammation.^[13]^ CGRP-related genes are good candidates to screen for an association with migraine susceptibility as several lines of evidence suggest that α-CGRP is a key player in migraine pathology. Plasma levels of CALCA have been found to be elevated during spontaneous and nitroglycerin-induced migraine attacks. However, in the present study we investigated a SNP at position −624(T/C) of the *CALCA* gene promoter (rs3781719), which may influence transcription of α-CGRP. CX3CL1 is a membrane-bound CX3C chemokine induced by primary proinflammatory signals in vascular endothelial cells (ECs).^[14]^ CX3CL1 is located at 16q13, it is a member of CX3C chemokines and plays an important role in various diseases.^[15]^ In the present study, we have selected CCL2 rs4586, CALCA rs3781719, and CX3CL1 rs614230 three SNPs, to study whether they contribute to the risk of chronic pain in Chinese Han women.

## 2 Materials and methods

### 2.1 Subjects

A total of 875 Chinese Han women who underwent cesarean section and lumbar-hard anesthesia were enrolled in our hospital during May 2014 and October 2017. Among them, 354 patients directly underwent cesarean section, 454 patients underwent cesarean section after trying delivery per vias naturalis, and 67 patients are first of second cesarean section. Twenty-five patients withdrew from the study, among them, the anesthesia effect of 21 patients could not complete the cesarean section, and it was necessary to assist local anesthesia or general anesthesia, 4 patients voluntarily withdrew from the study without any reason, and 850 patients remained. Among them, 350 women who developed chronic pain at 6 months after surgery were included as CPSP group, while 350 were randomly selected as a healthy control group (HC) among women who did not develop chronic pain at 6 months after surgery. In CPSP group, American Society of Anesthesiologists (ASA) grades I to II, age 20 to 39 years, mean age (28.5 ± 5.2) years old, weight 59 to 88 kg, BMI is (28.7 ± 2.7) kg/m^2^, gestational age 38 to 40 w. Exclusion criteria: maternal congenital cardiopulmonary disease, contraindications for spinal anesthesia, high blood pressure or gestational hypertension, hyperthyroidism or hypothyroidism, diabetes or gestational diabetes. The study was approved by the Medical Ethics Committee of Shanghai University of Medicine and Health Sciences Affiliated Zhoupu Hospital and The Affiliated Shanghai Pudong Hospital of Fudan University, and all subjects signed informed consent.

### 2.2 Anesthesia and postoperative analgesia

Anesthesia method was intrathecal injection of 10 mg ropivacaine and 4 μg of dexmedetomidine hydrochloride. The epidural analgesia was performed at L2–3 interspace by an 18-gauge Tuohy needle using the method of loss of resistance to air in left lateral position. After the epidural puncture was successful, the epidural puncture needle was used as a guide. A 25G lumbar puncture needle was inserted into the needle, and the arachnoid was pierced through the epidural puncture needle. The needle core was withdrawn and the cerebrospinal fluid was discharged from the needle. Injection of 0.5% ropivacaine plus 0.5 μg/mL dexmedetomidine 2 to 3 mL via spinal anesthesia, exit the lumbar puncture needle, and place the epidural catheter through the epidural needle (3 cm through the needle). Exit the epidural needle, the puncture point covers the sterile gauze, and the catheter was fixed. The spinal anesthesia block plane was measured after helping patients turn over and lie on his back. The epidural catheter was injected with 3 to 5 mL of local anesthetic, and after 5 min, there was no obstructed plane hyperactivity, and surgery can be started. During the operation, an appropriate amount of local anesthetic is injected through the epidural catheter as needed to maintain anesthesia. Analgesic method was 0.1% ropivacaine hydrochloride and 0.5 μg/kg dexmedetomidine hydrochloride were added to 0.9 mL physiological saline to 100 mL, and epidural continuous controlled analgesia was used. Maternal mean arterial pressure <60 mm Hg intravenous injection of ephedrine 10 mg, heart rate <50 bpm intravenous injection of atropine sulfate 0.2 mg. Epidural 10 mL of 1% lidocaine was administrated to rescue if VSA >3 after 30 min of epidural injection.

### 2.3 Genomic DNA isolation and PCR

**Figure 1:**
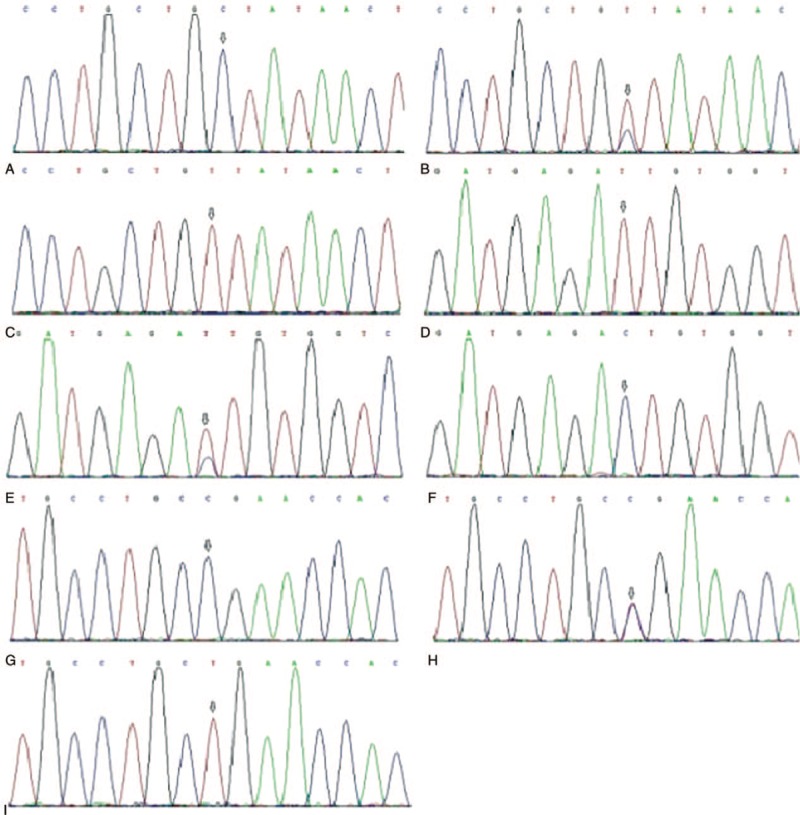
Sequencing chromatogram of mutations in CCL2 rs4586, CALCA rs3781719, and CX3CL1 rs614230. (A–C) are the result of genotyping of *CCL2* gene rs4586 locus, A is the CC genotype, B is the CT genotype, and C is the TT genotype. (D–F) are the result of CALCA rs3781719 site sequencing, D is the TT genotype, E is the TC genotype, and F is the CC genotype. (G–I) are the result of genotype sequencing of CX3CL1 rs614230 locus, where G is the CC genotype, H is the CT genotype, and I is the TT genotype.

We collected approximately 5 mL of the venous blood of each subject before the cesarean section, and then used the QIAamp DNA Blood Mini Kit (Qiagen, Germany) to obtain blood for genomic DNA analysis. After quantified by Nanodrop2000, DNA sequence was amplified using specific primers (Table 1). The PCR reaction system was as follows: 3 μL 10X rTaq buffer, 1 μL Forward primer, 1 μL Reverse primer, 2 μL dNTP, 2 μL gDNA, 0.2 μL rTaq, 20.8 μL ddH_2_O. PCR reaction conditions: pre-denaturation for 5 min at 95°C, denaturation for 30 s at 95°C, annealing for 45 s at 60°C, extension for 1 min at 72°C, 30 cycles, then another 10 min at 72°C. The PCR products were sent to complete the sequencing (Sunny, Shanghai), results were shown in Fig. 1.

**Table 1:**
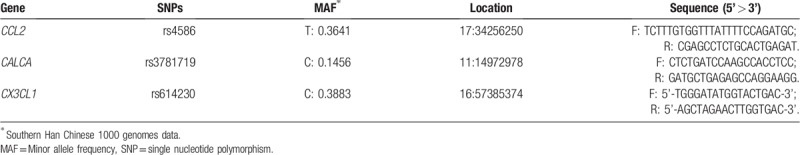
SNPs site primer sequence information.

### 2.4 Quantitative determination of CCL2, CALCA, and CX3CL1 in serum

The serum CCL2, CALCA, and CX3CL1 levels were detected by ELISA kit respectively (EKbio, Magden, Switzerland) strictly according to the manufacturer's instructions. Three replicate wells were tested at one time.

### 2.5 Statistical analysis

SPSS version 20.0 (IBM Corp., Armonk, NY, USA) was used for the statistical analysis. The descriptive statistics for quantitative variables were given as means standard deviation, comparison between the two groups were analyzed by *t* test. Differences between different genotypes were evaluated using one-way ANOVA. The allele frequencies and the distribution of genotypes were expressed as percentages, statistical analysis among multi-groups were performed by Chi-square test. Genotype frequency of two groups was checked by Chi-square test for Hardy–Weinberg equilibrium. The correlation between genotype and Chronic pain was analyzed by odds ratio (OR) and 95% confidence interval (CI), and factors such as age, BMI, gestational age, operation time, apgar score, and birth weight were corrected by multiple factor Logistic regression analysis. Logistic regression was performed to investigate association between SNP and CPSP. Generalized multifactor dimensionality reduction (GMDR)^[16]^ was used to analyze the interaction among three SNPs, and cross-validation consistency, the testing balanced accuracy, and the sign test, to assess each selected interaction, were calculated. Results were considered statistically significant when *P* values <.05.

## 3 Results

### 3.1 Genotype and allele distribution of mutations in *CCL2* gene

There was no significant difference between the two groups in age, BMI, gestational age, operation time, apgar score, and birth weight (Table 2). The comparison of genotype and allele frequencies of the *CCL2* gene rs4586 locus between the CPSP group and HC group was presented in Table 3. The genotype and allele distribution of *CCL2* gene rs4586 locus mutations in the control group were in Hardy–Weinberg equilibrium (*P* > .05). When wild-type CC was a reference, heterozygous (CT) and homozygous (TT) were protective factors for CPSP (adjusted OR = 0.812, 95%CI: 0.691–0.953, *P* = .010; adjusted OR = .584, 95%CI: 0.420–0.782, *P* < .001). CPSP risk were significantly reduced under both recessive and dominant model of *CCL2* gene rs4586 locus (adjusted OR = 0.755, 95%CI: 0.650–0.880, *P* < .001; adjusted OR = 0.641, 95%CI: 0.462–0.852, *P* = .001). The risk of CPSP in T allele carriers was significantly lower than that of C allele carriers (adjusted OR = 0.766, 95%CI: 0.675–0.865, *P* < .001) (Table 3).

**Table 2:**
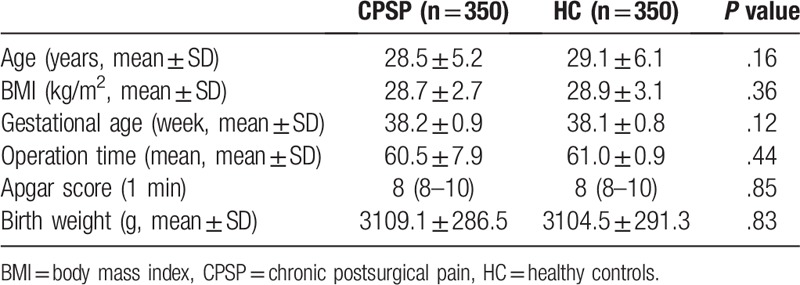
Basal characteristics of CPSP group and of HC group.

**Table 3:**
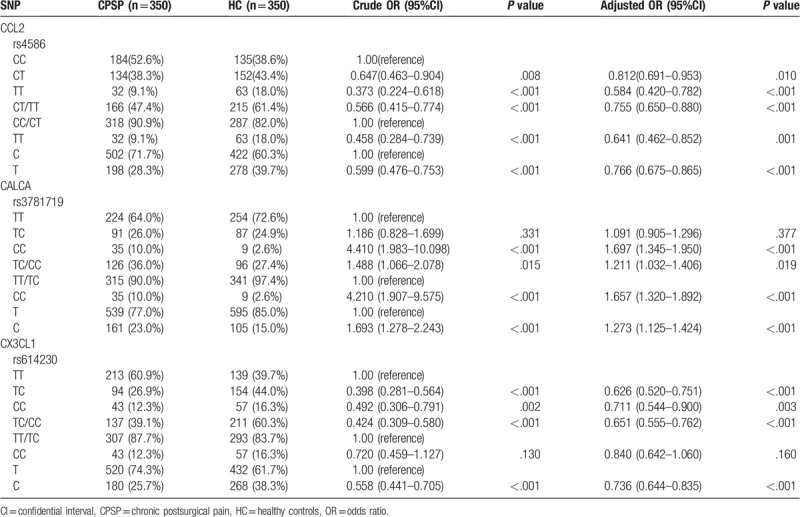
Genotype and allele frequency of *CCL2* rs4586, *CALCA* rs3781719, and *CX3CL1* rs614230 and the risk of CPSP analysis.

### 3.2 Genotype and allele distribution of mutations in *CALCA*

The genotype and allele distribution of *CALCA* gene rs3781719 locus mutations in the control group were in Hardy–Weinberg equilibrium (*P* > .05) (Table 3). In the case of wild type TT as a reference, homozygous (CC) was risk factors for CPSP (adjusted OR = 1.697, 95%CI: 1.345–1.950, *P* < .001), there is no significant change in the risk of CPSP in heterozygotes (adjusted OR = 1.091, 95%CI: 0.905–1.296, *P* = .377). CPSP risk were significantly increased under recessive and dominant model of *CALCA* gene rs3781719 locus (adjusted OR = 1.211, 95%CI: 1.032–1.406, *P* = 0.019; adjusted OR = 1.657, 95%CI: 1.320–1.892, *P* < .001). The risk of CPSP in C allele carriers was significantly higher than that of T allele carriers (adjusted OR = 1.273, 95%CI: 1.125–1.424, *P* < .001) (Table 3).

### 3.3 Genotype and allele distribution of mutations in *CX3CL1*

The genotype and allele distribution of *CX3CL1* gene rs614230 locus mutations in the control group were in Hardy–Weinberg equilibrium (*P* > .05) (Table 3). In the case of wild type TT as a reference, heterozygous (TC) and homozygous (CC) were protective factors for CPSP (adjusted OR = 0.626, 95%CI: 0.520–0.751, *P* < .001; adjusted OR = 0.711, 95%CI: 0.544–0.900, *P* = .003). CPSP risk were significantly decreased under recessive model of *CX3CL1* gene rs614230 locus (adjusted OR = 0.651, 95%CI: 0.555–0.762, *P* < .001). There was no significant change in the risk of CPSP under the recessive model (adjusted OR = 0.840, 95%CI: 0.642–1.060, *P* = .160). The risk of CPSP in C allele carriers was significantly lower than that of T allele carriers (adjusted OR = 0.736, 95%CI: 0.644–0.835, *P* < .001) (Table 3).

### 3.4 *CCL2*, *CALCA*, and *CX3CL1* gene–gene interaction

**Figure 2:**
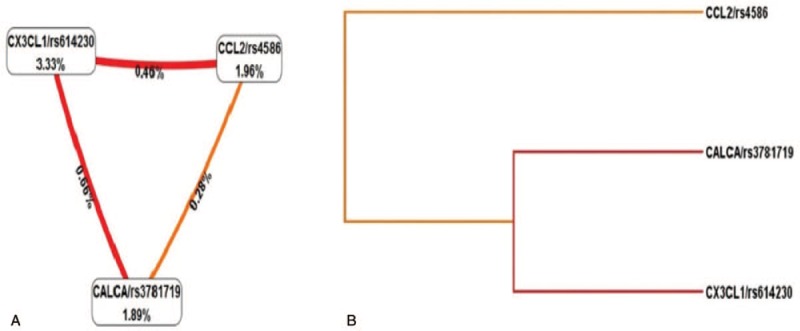
Hierarchical interaction graphs and interaction dendrogram. (A) Hierarchical interaction graphs showed that the percentage at the bottom of the each polymorphism represented entropy of it, and the percentage on each line represented the interaction percentage of entropy between the two SNPs. The red line represented synergy redundancy interaction and the orange line represented redundancy interaction. (B) Interaction dendrogram showed that the red line represented synergy interaction and the orange line represented synergy interaction more weakly. From left to right the interaction was more intensive.

**Figure 3:**
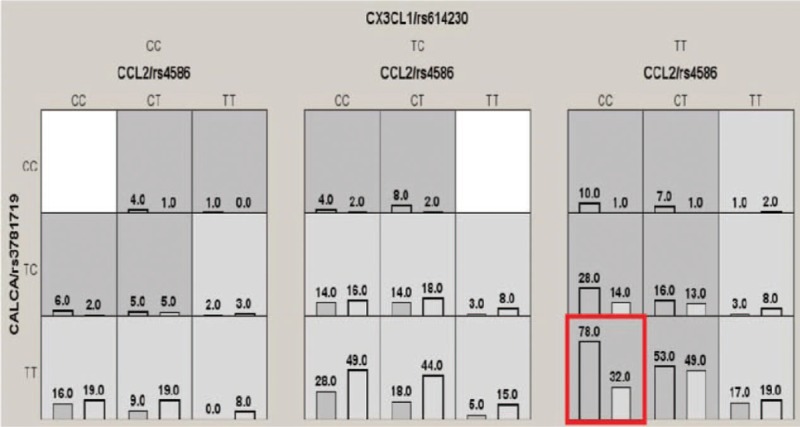
Distribution of high-risk and low-risk of hypertension in the best model. Dark gray and light gray boxes presented the high- and low-risk factor combinations, respectively. Left bars within each box represented chronic pain case while the right bars represented healthy control. The heights of the bars are proportional to the sum of samples in each group. Note that the patterns of high-risk and low-risk cells differ across each of the different multilocus dimensions. This is evidence of gene–gene interaction. The red box indicates the best combination for predicting CPSP risk.

We employed the GMDR analysis to investigate the impact of the interaction among three SNPs after adjustment for covariates. Table 4 summarizes the results obtained from GMDR analysis, and we found that there was a significant two-locus model (*P* < .001) involving *CALCA* gene rs3781719 locus and *CX3CL1* gene rs614230 locus. Indicating a potential gene–gene interaction between *CALCA* gene rs3781719 locus and *CX3CL1* gene rs614230 locus. Overall, the cross-validation consistency of this two-locus model was 10/10, and the testing accuracy was 62.71%. Hierarchical interaction graphs showed that the entropy of *CALCA* gene rs3781719 locus and *CX3CL1* gene rs614230 locus was 0.66%, which was higher other two interaction (Fig. 2A). Interaction dendrogram showed *CALCA* gene rs3781719 locus and *CX3CL1* gene rs614230 locus had a strong interaction (Fig. 2B). The best model of distribution of *CCL2*, *CALCA*, and *CX3CL1* gene–gene interaction was *CALCA* gene rs3781719 TT, *CCL2* gene rs4586 CC, and *CX3CL1* gene rs614230 TT genotype (Fig. 3).

**Table 4:**
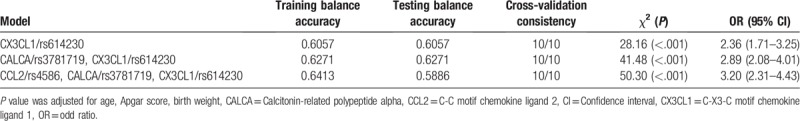
Summary of gene–gene interaction result in CPSP by GMDR.

### 3.5 The correlation between CCL2, CALCA, CX3CL1 protein levels and *CALCA*, *CCL2*, and *CX3CL1* gene polymorphism

**Figure 4:**
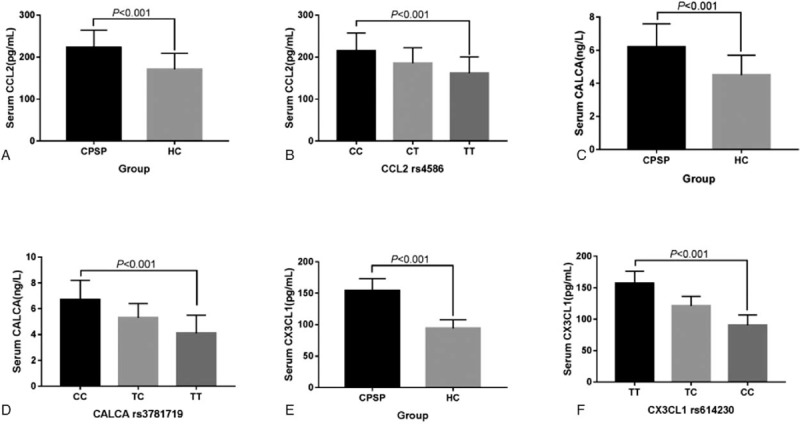
The comparison of CCL2, CALCA, and CX3CL1 protein levels. CPSP represents chronic postsurgical pain. HC = healthy control.

We used ELISA to detect the levels of CCL2, CALCA, and CX3CL1 in serum of all subjects. The results showed that the serum levels of CCL2, CALCA, and CX3CL1 in women with chronic pain after cesarean section were significantly higher than those in healthy controls, the difference were statistically significant (*P* < .001) (Fig. 4A, C, E). The levels of CCL2, CALCA, and CX3CL1 in serum of different genotypes were analyzed. The results showed that the *CCL2* rs4586 locus CC genotype had the highest serum CCL2 protein level, the CT genotype was the second, followed by CT and TT (*P* < .001) (Fig. 4B). The *CALCA* gene rs3781719 locus TT genotype had the highest serum CALCA level, followed by CT and TT (Fig. 4D). Subjects carrying the *CX3CL1* gene rs614230 locus TT genotype had the highest serum CX3CL1 levels, followed by TC and CC (Fig. 4F).

## 4 Discussion

In this study, we analyzed the relationship between chronic pain after cesarean section in Chinese Han women and *CCL2* gene rs4586, *CALCA* gene rs3781719 locus, and *CX3CL1* gene rs614230 locus single nucleotide polymorphism, and found *CCL2* gene rs4586, *CALCA* rs3781719, *CX3CL1* rs614230 gene polymorphism were associated with the risk of CPSP in Chinese Han women.

Pain, tension, and anxiety are common problems after cesarean section, which can affect maternal eating and rest, and thus affect rehabilitation and lactation. Clinical studies have found that the incidence of chronic pain after cesarean section is 15% at 3 months to 11% at 12 months or longer.^[17]^

The causes of CPSP and its relationship with acute pain are still not clear. The possible mechanisms include: pain amplification mechanism caused by peripheral and central sensitization, inhibitory regulation of central nervous system and sympathetic nervous system regulation mechanism.^[7]^ Inflammatory reactions, especially peripheral and central nervous system inflammation, play an important role in the formation of CPSP.^[18]^ The repair process of peripheral tissue injury after operation includes three stages: inflammatory reaction, inflammatory cell infiltration, and tissue remolding. The pain caused by acute inflammatory response is mainly caused by the release of inflammatory neuropeptides, including substance P, calcitonin gene-related peptide (CGRP), and C-nerve fiber neurokinin A (NKA); all of them are important priming factors in the process of neural system remodeling.^[19]^ Acute surgical trauma can lead to severe pain and pain hypersensitivity, but it usually relieves as the wound heals. The duration of acute inflammatory reaction is related to wound healing speed and the patient's basic physical condition, which lasts for 24 h to 2 weeks. When the wound repair is not good, the chronic inflammatory reaction in the injured site of hand operation can persist, leading to the occurrence of CPSP.^[20]^

The *CCL2* gene is located in 17q12 and consists of three exons and two introns. There is a proximal and a distal regulatory regions in the *CCL2* gene, both located upstream of the transcription start point, and the proximal regulatory region can bind to transcription factors, affecting the level of transcription of the gene.^[21]^ The distal regulatory region contains a NF-κB binding site necessary for cytokine to have *CCL2* expression.^[22]^*CCL2* gene rs4586 is located in the second exon, and this mutation is associated with the occurrence of various diseases. For example, Sunakawa et al^[23]^ showed that *CCL2* gene rs4586 is associated with gastric cancer progression. We believe that this mutation may affect the expression of *CCL2* protein, because from the results of this study, the level of CCL2 protein in the serum of mutant individuals is obviously higher, and the corresponding clinical symptoms show that the risk of CPSP in mutant individuals is lower. However, it is not yet fully confirmed that *CCL2* gene rs4586 locus SNP affects the expression of CCL2 protein and leads to the occurrence and development of CPSP, because we lack cell model validation. The level of CCL2 protein in serum does not fully reflect the expression level of this protein, as other factors may influence the level of CCL2 protein in serum.^[24]^

The *CALCA* gene encodes calcitonin gene-related peptide (CGRP), which is an important priming factor in the process of neural system remodeling. Lassen et al^[25]^ found that the increase in CGRP observed during the onset of spontaneous migraine suggests that CGRP may play a role in the pathogenesis of spontaneous migraine. Our study also showed that CGRP levels were higher in the CPSP group, which is consistent with the results of Lassen's study. The CALCA level of *CALCA* gene rs3781719 site mutation (CC) carriers was significantly higher than that of other genotype carriers. This phenomenon suggests that *CALCA* gene rs3781719 site mutation may be the intrinsic cause of CALCA expression level change, and further research is needed.

Chemokine (C-X3-C motif) ligand 1 gene (*CX3CL1*, also known as fractalkine), located at 16q13, is a member of CX3C chemokines and plays an important role in various diseases.^[15,26–28]^*CX3CL1* rs614230 gene SNP is a disease-related site, and Jin et al^[29]^ showed that the change of point SNP was associated with coronary artery disease (CAD). The results of this study showed that this mutant was a protective factor for chronic pain. The serum CX3CL1 level in mutants was also significantly lower than that in wild-type. We speculate that this correlation may be the cause of CPSP. The wild-type individual has a higher level of CX3CL1, which is a pro-inflammatory cytokines can act directly or indirectly on noxious sensory receptors, activation of many complex signal transduction pathways, such as protein kinase A, protein excitase C, p38 mitogen-activated protein kinase (MAPK), in order to reduce the excitatory threshold of peripheral neurons and produce short-term peripheral sensitization.^[8]^

In addition, we also studied whether *CCL2*, *CALCA*, and *CX3CL1* genes have synergistic effects on CPSP, and the results show that there is a potential gene–gene interaction between the *CALCA* gene rs3781719 locus and the *CX3CL1* gene rs614230 locus. Gene–gene interactions and gene–environment interactions may be major factors influencing the occurrence of CPSP. The role of a single gene may be very limited, so screening for more genes and related environmental factors, and analyzing the effects of gene–gene and gene–environmental factors may be very meaningful.

The results of our study suggest an association between *CCL2*, *CALCA*, and *CX3CL1* gene polymorphisms and CPSP. Nevertheless, this hypothesis requires further investigation, and it may be necessary to expand the sample size and set up in vitro cell experiments.

## 5 Conclusion

*CCL2* gene rs4586, *CALCA* rs3781719, *CX3CL1* rs614230 gene polymorphism are associated with the occurrence of chronic pain after cesarean section in Chinese Han women.

## Author contributions

**Formal analysis:** Chengquan Huang.

**Investigation:** Rui Wang.

**Methodology:** Guoping Ma, Bange Zhao, Rui Wang.

**Resources:** Guoping Ma, Jingli Yang.

**Software:** Jingli Yang.

**Supervision:** Guoping Ma.

**Validation:** Bange Zhao, Chengquan Huang.

**Visualization:** Rui Wang.

**Writing – original draft:** Guoping Ma.

**Writing – review & editing:** Guoping Ma.
